# From yeast to humans: Understanding the biology of DNA Damage Response (DDR) kinases

**DOI:** 10.1590/1678-4685-GMB-2019-0071

**Published:** 2019-12-13

**Authors:** José Renato Rosa Cussiol, Bárbara Luísa Soares, Francisco Meirelles Bastos de Oliveira

**Affiliations:** 1 Departamento de Bioquímica, Universidade Federal de São Paulo, São Paulo, SP, Brazil; 2 Instituto de Biofísica Carlos Chagas Filho, Universidade Federal do Rio de Janeiro, Rio de Janeiro, RJ, Brazil

**Keywords:** Yeast, genome instability, DNA damage response, cell cycle checkpoint, kinase

## Abstract

The DNA Damage Response (DDR) is a complex network of biological processes that protect cells from accumulating aberrant DNA structures, thereby maintaining genomic stability and, as a consequence, preventing the development of cancer and other diseases. The DDR pathway is coordinated by a signaling cascade mediated by the PI3K-like kinases (PIKK) ATM and ATR and by their downstream kinases CHK2 and CHK1, respectively. Together, these kinases regulate several aspects of the cellular program in response to genomic stress. Much of our understanding of these kinases came from studies performed in the 1990s using yeast as a model organism. The purpose of this review is to present a historical perspective on the discovery of the DDR kinases in yeast and the importance of this model for the identification and functional understanding of their mammalian orthologues.

## Introduction

Despite its apparent stability, DNA can undergo significant changes in its structure. Spontaneous hydrolysis, oxidation and non-enzymatic methylation of DNA nitrogen bases can induce tens of thousands of lesions per day (Lindahl and Nyberg, 1972; Lindahl, 1993). In addition, environmental agents such as genotoxic chemicals, ultraviolet light (UV) and ionizing radiation (IR) can increase the frequency of single strand breaks (SSBs) and double strand breaks (DSBs) (Friedberg, 2008; Giglia-Mari *et al.*, 2011). Replicating cells are particularly susceptible to DNA lesions because the progression of replication forks can be hampered by DNA adducts, DNA-RNA hybrids, protein-DNA complexes or depletion of dNTP pools (Lambert and Carr, 2013). These lesions, if left unrepaired, can lead to genomic instability, which is a hallmark of cancer and other diseases. Thus, eukaryotic cells regulate a set of biological processes collectively entitled as DNA Damage Response (DDR) (Ciccia and Elledge, 2010).

The DDR comprises multiple DNA repair and DNA damage tolerance pathways, as well as cell cycle checkpoints. Therefore, the existence of a DNA damage signaling pathway responsible for ensuring efficient, accurate and timely DDR is imperative for cell survival. One of the most important layers of DDR regulation comprises a complex signaling network mediated by serine/threonine kinases members of the phosphatidylinositol-3-kinase-like kinase family (PI3K-like or PIKKs). In mammals, this signaling network is orchestrated by the DDR kinases ATR, ATM and DNA-PK (Blackford and Jackson, 2017). These kinases act as DNA damage sensors and effectors, recognizing alterations in the DNA molecule and eliciting a signaling cascade through the phosphorylation of hundreds of proteins (Falck *et al.*, 2005; Marechal and Zou, 2013).

Since the discovery of the DDR kinases, much progress has been made in the understanding of their role in genome stability. Due to its biological relevance, DDR is highly conserved from yeast to humans (Table 1). Of note, the identification and dissection of the molecular function of the DDR kinases was only possible because of the contribution of several laboratories studying DNA damage signaling in model organisms such as *Saccharomyces cerevisiae and Schizosaccharomyces pombe*. Therefore, yeast has been placed as an attractive model to uncover the molecular mechanisms behind the function of the DDR kinases. In this review, we offer a historical perspective of the identification and characterization of DDR kinases by following the chronology of classical studies and highlighting their contributions to the understanding of the DDR pathway.

**Table 1:** DDR kinases homologs in yeast and human.

*S. cerevisiae*	*S. pombe*	Human
*MEC1*	*rad3*	*ATR*
*TEL1*	*tel1* *	*ATM*
*RAD53*	*cds1*	*CHK2*
*CHK1*	*chk1*	*CHK1*
*DUN1*	-	-
-	-	*DNA-PK*

* (Naito *et al.*, 1998)

## Dun1, the first kinase associated with DDR in eukaryotes

**Figure 1:**
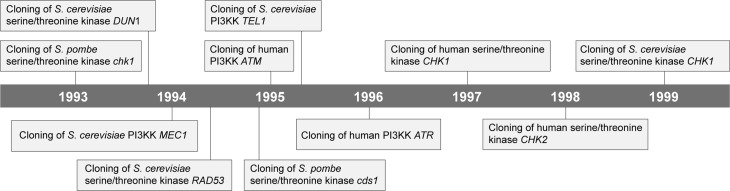
Timeline for the discovery of DDR kinases in yeast and human. Although the gene encoding *S. pombe* Chk1 was identified before Dun1, its kinase activity was associated with DDR only in 1996 (Walworth and Bernards, 1996).

**Figure 2:**
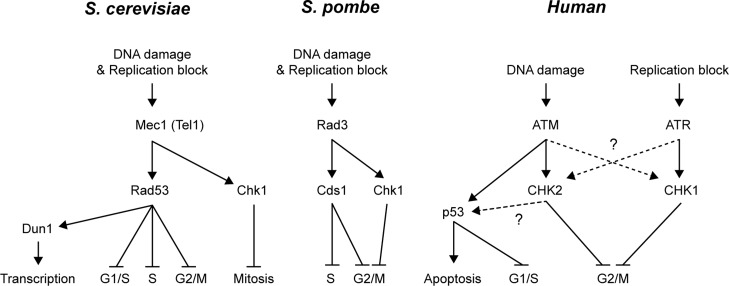
Schematic representation of the signaling network of DDR kinases in yeast and human. In *S. cerevisiae*, DNA damage and replication block signal is preferentially transduced from Mec1 to Rad53 and Chk1 with Tel1 showing an overlap with Mec1. Rad53 inhibits the G1/S, S phase and G2/M cell cycle transitions and activates transcription in a Dun1-dependent manner. Chk1 acts in parallel to Rad53 inhibiting mitosis by preventing anaphase entry. In *S. pombe*, DNA damage and replication block signal is preferentially transduced from Rad3 to Cds1 and Chk1. Cds1 inhibits S phase and reinforces G2/M inhibition together with Chk1. In mammalian cells, ATM signals to p53, which in turn activates apoptosis and inhibits G1/S cell cycle transition (Kastan *et al.*, 1991; Lowe *et al.*, 1993). While ATM signals to CHK2, ATR signals to CHK1 in response to DNA replication inhibition. Both CHK1 and CHK2 inhibit the G2/M cell cycle transition, although at that time their roles during S phase progression were unknown. Also, there was still no evidence on the crosstalk between ATM/ATR with CHK1/CHK2, nor between CHK2 and p53. Dashed lines and interrogation marks represent unknown pathways at that time.

In the late 1980s, in an attempt to identify a recombinase in *S. cerevisiae*, Stephen Elledge accidentally isolated the gene encoding a subunit of ribonucleotide reductase (*RNR2*) (Elledge and Davis, 1987; Elledge, 2015). The initial disappointment, however, turned into curiosity when *RNR2* expression was shown to be dependent on treatment with drugs that interfere with DNA replication (Elledge and Davis, 1987, 1989). This result suggested that eukaryotic cells could modulate nucleotide synthesis in response to DNA damage caused during replication arrest. Reinforcing this hypothesis, in the following years, genes encoding other subunits of the ribonucleotide reductase such as *RNR1* and *RNR3* were isolated, both presenting expression patterns similar to those observed for *RNR2* (Elledge and Davis, 1990). In the early 90s, in order to understand the molecular basis of this signaling mechanism, the Elledge laboratory developed a genetic screen to identify genes involved in the regulation of *RNR3* expression. The approach aimed to identify mutants of *S. cerevisiae* that repressed *RNR3* expression upon treatment with hydroxyurea (HU), a DNA synthesis inhibitor. Mutants isolated in this screen were referred to as DNA-damage uninducible (*dun*) (Zhou and Elledge, 1993). Among the isolated candidates the most promising was a serine/threonine protein kinase named Dun1 (Figure 1). The sensitivity of *dun1* mutants to HU suggested that upregulation of ribonucleotide reductase was an important event for cell tolerance to DNA replication arrest. Most importantly, this observation reinforced the existence of a signaling pathway responding to DNA damage in eukaryotic cells. Indeed, metabolic labeling with ^32^P-labeled phosphate showed that Dun1 became highly auto-phosphorylated in response to HU, suggesting that its function was actively modulated during DDR (Zhou and Elledge, 1993). In addition to autophosphorylation, Dun1 presented another phosphorylated form that occurred independently of its catalytic activity. Although its function was not clear, this raised the possibility that upstream kinases might be involved in regulating this signaling pathway (Figure 2). Curiously, although *dun1* mutants showed reduced expression of ribonucleotide reductase, the cell cycle checkpoints remained intact (Zhou and Elledge, 1993). This suggested a possible ramification of the DDR in *S. cerevisiae* where, in addition to Dun1, other signaling components were required to regulate different functions necessary to protect cells against damage arising during DNA replication (Figure 2).

## Mec1, a yeast PI3K-like kinase linking cell cycle checkpoints and meiotic recombination

In the early 90s, Lee Hartwell and Ted Weinert observed that the combination of mitotic checkpoint mutant *rad9* (radiation sensitive 9) with *cdc13* (cell division cycle 13)*,* showed a striking loss of viability when compared to single mutants alone (Weinert and Hartwell, 1993). *cdc13* was defective for telomere metabolism, accumulating aberrant DNA structures near the end of the chromosomes. The authors inferred that loss of viability of the double mutant could be attributed to cell division with aberrant DNA structures. Based on the genetic interaction observed for *cdc13 rad9*, Lee Hartwell and Ted Weinert developed a screen to identify new genes involved in the regulation of the mitotic checkpoint. By inducing random mutations in a *cdc13* mutant and analyzing more than 12,000 strains, the authors identified four mutants with a strong negative genetic interaction. These mutants were named mitosis entry checkpoint (*mec*) and included *mec1*, *mec2*, *mec3* and *rad9* itself (Weinert *et al.*, 1994) (Figure 1). Less than one year later, *mec1* was also identified as *sad3* and *esr1* by two independent research groups. In the first case, *sad3* was identified by Stephen Elledge’s group in a screen performed to identify HU-sensitive mutants. These mutants were named S-phase arrest-defective (*sad*) because, in addition to mitotic checkpoint defects, they were also defective in the S phase checkpoint (Allen *et al.*, 1994). Reinforcing this finding, Paulovich and Hartwell (1995) demonstrated that slowing of replication forks during DNA damage is an active process dependent on *MEC1* .

Based on the hypothesis that meiosis II was similar to a mitotic division, Ryuichi Kato and Hideyuki Ogawa performed a screen to identify mutants that were not only sensitive to DNA damage agents but also defective in meiotic recombination. Using this approach the authors identified and cloned an essential gene required for DNA repair and meiotic recombination named *ESR1* (Kato and Ogawa, 1994). Interestingly, the *ESR1*-encoded protein showed high similarity with phosphatidylinositol 3-kinases (PI3K). However, at that time it was unclear whether Esr1-mediated signal transduction was restricted to lipid phosphorylation.

As described in the following sections, the discovery of other kinases associated with the DDR reinforced functional and structural divergences between Esr1 and classical PI3Ks (Keith and Schreiber, 1995). For this reason, together with Esr1, these kinases were then referred to as PI3K-like protein kinase (PIKKs) For the purpose of this review and following the chronology of identification, henceforth, *MEC1*/*ESR1*/*SAD3* will be referred to as *MEC1*.

## Tel1, the yeast ortholog of ATM, suggests the existence of parallel pathways in the DDR

Ataxia telangiectasia (A-T) is an autosomal recessive syndrome characterized by neurodegeneration, immunodeficiency and cancer predisposition. Cells derived from A-T patients show genomic instability and are highly sensitive to IR (Shiloh and Rotman, 1996). In the early 1990s, the main hypothesis for this phenotype suggested a dysfunctional cell cycle checkpoint (Beamish *et al.*, 1994). Over more than five years, the extensive work of several research groups helped to narrow down the genomic region containing the defective gene potentially associated with the A-T phenotype (Gatti *et al.*, 1988; McConville *et al.*, 1994; Rotman *et al.*, 1994; Lange *et al.*, 1995). Finally, in 1995, a consortium lead by Yosef Shiloh’s laboratory cloned the *ATM* (ataxia telangiectasia mutated) gene and identified its respective mutations in A-T patients (Savitsky *et al.*, 1995a,b) (Figure 1).

The amino acid sequence encoded by *ATM* showed similarity with the PIKK Mec1 cloned a few months earlier in *S. cerevisiae* (Keith and Schreiber, 1995; Savitsky *et al.*, 1995a). In addition to Mec1, ATM also showed strong similarity to the amino acid sequence encoded by the *S. cerevisiae* open reading frame (ORF) *YBL088* (Savitsky *et al.*, 1995b)*.* At that time, two research groups independently identified and cloned the gene correspondent to *YBL088* (Greenwell *et al.*, 1995; Morrow *et al.*, 1995). Interested in understanding the mechanisms that led to telomere maintenance defects in the *tel1* mutant, Greenwell *et al.* (1995) cloned a DNA fragment containing *TEL1*, and interestingly, the analysis of the amino acid sequence encoded by *TEL1* was identical to the product of *YBL088* (Figure 1). At the same time, surprised by the enormous similarity between the amino acids sequences encoded by *ATM* and *YBL088*, Morrow *et al.* (1995) cloned the gene correspondent to *YBL088* and, aware of the parallel work of Greenwell, referred to the gene also as *TEL1* (Figure 1). Corroborating the functional conservation between *ATM* and *TEL1*, *tel1* mutants showed an increase in the frequency of mitotic recombination and loss of chromosomes similar to that observed in A-T cells (Greenwell *et al.*, 1995). However, *tel1* mutants showed no sensitivity to genotoxic agents, suggesting the existence of parallel pathways that could bypass Tel1 function upon DNA damage conditions (Greenwell *et al.*, 1995). Indeed, the similarities between the primary structures of Mec1 and Tel1 suggested a functional overlap between these two proteins (Morrow *et al.*, 1995) (Figure 2). The authors confirmed this, in part, by showing that an extra copy of *TEL1* was able to partially rescue the sensitivity in Mec1-deficient cells treated with IR, UV or HU (Morrow *et al.*, 1995). Interestingly, although *mec1* mutants were more sensitive to genotoxic agents than *tel1*, they did not display deficiencies in telomere maintenance (Greenwell *et al.*, 1995; Morrow *et al.*, 1995). This suggested that despite the functional overlap, these kinases could also have functions dependent on the type of DNA damage: Mec1 would be preferentially related to the response to DNA damage induced by IR, UV and HU while Tel1 would be involved in the response associated with damaged telomeres. Although Mec1 and ATM dysfunctions were phenotypically similar, ATM had greater similarity to Tel1. Therefore, it was plausible to infer the existence of a *MEC1* ortholog capable of exerting parallel functions to *ATM* in human cells (Figure 2).

## ATR, the human ortholog of Mec1, has a role in the response to DNA damage caused during DNA replication

*rad3* of *Schizosaccharomyces pombe* was previously identified as a radiation sensitive mutant with defects in the cell cycle checkpoints (al-Khodairy and Carr, 1992; Jimenez *et al.*, 1992). A few years after the characterization of *rad3*, Savitsky *et al.* (1995a) demonstrated a high similarity between ATM, Mec1 and a partial sequence of Rad3. Motivated by this similarity, Tony Carr’s laboratory isolated the coding region corresponding to Rad3 and demonstrated the presence of C-terminus consensus sequences that defined Rad3 as a new member of the PIKK family (Bentley *et al.*, 1996) (Figure 1). They also demonstrated that a kinase-dead mutant of Rad3 recapitulated the phenotypes described for its null mutant, suggesting that kinase activity was essential for Rad3 function. In addition, Rad3 immunoprecipitates were shown to exhibit an associated protein kinase activity, supporting the ability of PIKK to catalyze the phosphorylation of protein substrates (Bentley *et al.*, 1996).

Although belonging to the same family of PIKK, sequence analysis of Rad3, Mec1, Tel1 and ATM suggested an evolutionary divergence in two distinct subfamilies. One subfamily comprised of Rad3/Mec1 and the other of Tel1/ATM (Table 1). However, unlike the human *ATM,* which is closely related to *TEL1*, the existence of a human ortholog for *MEC1* and *rad3* remained unknown. In an attempt to identify the human ortholog of *rad3*, Tony Carr’s laboratory used degenerated PCR based on the sequences of Rad3 and Mec1 and subsequently screened a cDNA library isolating the coding region of *ATR* (ataxia telangiectasia and *r* *ad3*-related) (Bentley *et al.*, 1996) (Figure 1). At the same time, Cimprich *et al.* (1996) relied on an expressed sequence tag (EST) with sequence similarity to the PI3K-related kinases FRAP, Tor1p and Tor2p to isolate the cDNA corresponding to *FRP1* (FRAP-related protein 1). *FRP1* sequence was shown to be identical to that of *ATR* and, by convention, was referred to by the same name (Figure 1).

ATR presented elements that characterized it as a PIKK showing higher similarity to Rad3/Mec1 than to ATM/Tel1 (Keith and Schreiber, 1995; Bentley *et al.*, 1996). In addition, the overexpression of *ATR* was able to rescue the sensitivity of a *mec1* partial defective mutant, demonstrating a functional conservation between these kinases (Bentley *et al.*, 1996). These observations reinforced the idea that *ATR* was the human ortholog of *MEC1*/*rad3* while *ATM* was the ortholog of *TEL1* (Table 1 and Figure 2).

Previous comparative studies between Mec1 and Tel1 suggested a possible ramification of the DDR pathway, where the different kinases would respond to different types of DNA insults (Morrow *et al.*, 1995). Identification of ATR and ATM suggested that, as in yeast, these kinases would also perform specialized functions in mammalian cells. However, due to the absence of an available model to mimic ATR defects, functional studies on this kinase were only possible in 1998, when Cliby *et al.* (1998) developed a dominant negative mutant based on the overexpression of a kinase-dead allele of *ATR*. These experiments confirmed that, unlike cells of A-T patients that exhibit sensitivity to a narrow range of DNA-damaging agents, ATR inactivation promoted sensitivity to various types of agents, including those affecting DNA replication (Cliby *et al.*, 1998). Therefore, as previously suggested, it was demonstrated that despite a functional overlap with ATM, ATR was preferentially involved in the response to DNA damage caused during DNA replication. It is now clear that ATM recognizes DSBs by association with the MRN complex, whereas ATR recognizes RPA-coated single-stranded DNA, a byproduct of multiple DNA damage and replication arrest agents (Blackford and Jackson, 2017).

## Rad53, a *S. cerevisiae* protein kinase with a central role in DDR

During inhibition of DNA replication, the S phase checkpoint promotes an arrest of the cell cycle prior to mitosis. To understand the mechanisms regulating this checkpoint, Stephen Elledge’s laboratory developed a screen to identify yeast mutants that were sensitive to HU. As mentioned before, mutants isolated in this screen were referred to as *sad* (S-phase arrest-defective) and included *sad1 to sad5.* (Allen *et al.*, 1994). Among the isolated mutants, *sad1* had the highest sensitivity to HU and therefore was selected for further investigation. In addition to presenting dysfunctional S phase checkpoint, *sad1* mutants had defects in the G1 and G2/M checkpoints (Allen *et al.*, 1994). Interestingly, inhibition of Cks1, a regulatory subunit of cyclin-dependent kinase Cdc28, rescued *sad1* sensitivity to HU (Allen *et al.*, 1994). Considering that Cks1 activity was required for both G1/S and G2/M transitions, it was suggested that in response to DNA damage, Sad1 would negatively regulate Cks1 to prevent its cell cycle transition-promoting activity. Furthermore, *sad1* mutants showed a reduction in Dun1 phosphorylation levels associated with a decrease in the expression of *RNR2* and *RNR3* (Allen *et al.*, 1994). Interestingly, as mentioned in previous sections of this review, while it was demonstrated that Dun1 was required for the transcriptional response to DNA damage, it was not required to control cell cycle checkpoints (Zhou and Elledge, 1993). Considering that *sad1* mutants were defective for both cell cycle arrest and *RNR* expression, it was suggested that Sad1 functions upstream of Dun1 in the signaling pathway responsible for the DNA damage transcriptional response (Figure 2). *SAD1* was cloned by complementation assays and its coding region was shown to be identical to *SPK1*, a previously isolated gene encoding a serine/threonine protein kinase (Stern *et al.*, 1991) (Figure 1). *sad1* was also shown to be allelic to *rad53*, a radiation-sensitive mutant identified in 1974 (Game and Mortimer, 1974). Eventually it was shown that both mutants had the same defective gene and therefore *SAD1* was referred to as *RAD53* (Allen *et al.*, 1994).

In order to identify genes involved in the regulation of Rad53, Sanchez *et al.* (1996) performed a screen to identify mutants whose viability depended on the overexpression of Rad53. Surprisingly, one of the isolated mutants was *mec1* (Kato and Ogawa, 1994; Weinert *et al.*, 1994) (Figure 1). Supporting the functional dependency between Mec1 and Rad53, Mec1 was also shown to be involved in the regulation of the S phase checkpoint (Allen *et al.*, 1994; Weinert *et al.*, 1994; Paulovich and Hartwell, 1995). In addition to salvaging the lethality of a *mec1* null mutant, the overexpression of *RAD53* rescued the HU and UV sensitivity of a *mec1* partial mutant (Sanchez *et al.*, 1996). Together, these observations suggested that Rad53 mediated the essential function of Mec1 and therefore, acted downstream of Mec1 in the DDR pathway (Figure 2). Using a phospho-dependent electrophoretic mobility shift assay the authors showed that Rad53 was phosphorylated in response to HU and methyl methanesulfonate (MMS) treatment, demonstrating that its function was actively modulated during DNA replication arrest and DNA damage. The authors also showed that Rad53 phosphorylation decreased in *mec1* defective mutants. Interestingly, an extra copy of *TEL1* partially complemented Rad53 phosphorylation levels, reinforcing the idea of a functional overlap between Mec1 and Tel1 (Sanchez *et al.*, 1996) (Figure 2).

The identification and cloning of *RAD53* helped to integrate the functions of Mec1 and Tel1 in the regulation of cell cycle checkpoints and dNTPs synthesis (Figure 2). At that point, Mec1, Tel1, Rad53 and Dun1 constituted the central components of a signaling pathway whose phosphorylation cascade would ultimately regulate the DDR (Figure 2). It is important to note that, unlike Dun1, whose transcriptional function in inducing RNR activity is substituted by the transactional factor p53 in human cells (Kastan *et al.*, 1992), Mec1 and Tel1 are orthologs of ATR and ATM, respectively (Table 1 and Figure 2).

## Chk1 and Cds1 kinases of *S. pombe*: dual regulators of the DDR

In addition to studies in *S. cerevisiae*, studies in *S. pombe* had an important contribution in the identification and functional characterization of DDR kinases. In the early 1990’s it was known that cell cycle transition to mitosis depended largely on *cdc2* (cell division cycle 2). To better understand the mechanisms involved in the regulation of Cdc2, Walworth *et al.* (1993) introduced a multicopy gene library into a temperature-sensitive *cdc2* mutant and isolated plasmids that allowed the cells to grow at restrictive temperatures. One of these plasmids carried a gene encoding a serine/threonine kinase referred to as *chk1* (checkpoint kinase 1) (Figure 1). Almost at the same time, al-Khodaire *et al.* (1994) isolated a gene that complemented the checkpoint defects of a *rad27* (radiation sensitive 27) mutant. This gene was found to be identical to the *chk1* gene previously isolated by Walworth *et al.* (1993) and, by convention, it was referred to by the same name.

Supporting the role of Chk1 in cell cycle arrest, *chk1* null mutants presented mitotic checkpoint defects and increased sensitivity in response to UV and IR treatments (Walworth *et al.*, 1993; al-Khodairy *et al.*, 1994). Chk1 was phosphorylated in response to UV, MMS and IR treatments, suggesting that the kinase was actively regulated during DDR. In addition, it was demonstrated that a kinase-dead mutant of Chk1 was more sensitive to UV than the wild type, implying that its kinase activity was also important for cellular response to DNA damage. Further experiments showed a reduction on Chk1 phosphorylation in several *rad* mutants, including *rad3* (Walworth and Bernards, 1996). These observations suggested that in *S. pombe*, Rad3 acted upstream to Chk1 in the regulation of mitotic checkpoint (Figure 2). Interestingly, *chk1* null mutants displayed hypersensitivity and mitotic checkpoint defects when treated with UV, MMS and IR but not with HU. Moreover, Chk1 did not undergo changes in phosphorylation levels in HU-treated cells (Walworth and Bernards, 1996).

In 1995, the serine/threonine kinase Cds1 (checking DNA synthesis 1) was identified as a suppressor of a temperature-sensitive mutant of polymerase α (Murakami and Okayama, 1995) (Figure 1). *cds1* null mutants showed loss of viability associated with S phase entry in the presence of HU. Supporting its role during DNA replication, Cds1 was phosphorylated and activated in response to HU. Additionally, *cds1* null mutants lost the ability to slow S phase in HU-treated cells (Lindsay *et al.*, 1998). This observation suggested that while Chk1 responded to DNA damage, Cds1 preferentially responded to DNA replication arrest. However, in *cds1* null mutants Chk1 could be activated in response to HU treatment, suggesting that this dynamic was not so simplistic. In fact, *chk1 cds1* double mutants were more sensitive to HU than single mutants alone, suggesting some functional overlap between the two kinases (Boddy *et al.*, 1998; Lindsay *et al.*, 1998). Corroborating these observations, Paul Russell’s laboratory showed that both Chk1 and Cds1 regulate Cdc2, the kinase responsible for mitosis initiation. While Chk1 and Cds1 inhibit Cdc25 indirectly by repressing Cdc2, Cds1 was required to increase the abundance of Mik1, a Cdc2 repressor (Furnari *et al.*, 1997; Rhind *et al.*, 1997; Boddy *et al.*, 1998; Baber-Furnari *et al.*, 2000; Christensen *et al.*, 2000). Thus, although Cds1 and Chk1 regulate sub pathways of Rad3 response, both Cds1 and Chk1 (Figure 2) are able to control the mitotic checkpoint in response to DNA replication arrest.

Despite the structural similarity, Cds1 and Rad53 show marked differences in their functions throughout the cell cycle (Figure 2). While Rad53 has a broader role, acting along G1, S and G2 phases, Cds1 acts only during S phase (Figure 2). In this context, researchers have directed their attention to the existence of a potential mammalian counterpart of Rad53 and Chk1, which could aid understanding of how mammalian cells transduce their signals to regulate DDR.

## CHK1 and CHK2: linking DNA damage to cell cycle checkpoints in mammalian cells

To identify the human ortholog of *S. cerevisiae RAD53* and *S. pombe cds1*, Matsuoka *et al.* (1998) used the information of an EST with sequence similarity to the conserved FHA domain of Rad53 and Cds1 to screen a human cDNA library. By using this strategy, the authors isolated the cDNA encoding a protein with 26% identity with both Rad53 and Cds1. This gene was name *CHK2* (checkpoint kinase) in reference to *CHK1*, a gene of similar function identified a few months earlier by the same research group (Sanchez *et al.*, 1997) (Figure 1). Supporting functional conservation with *RAD53*, the expression of *CHK2* complemented the lethality of *rad53* null mutants. In addition, as demonstrated for Rad53, cells exposed to UV or IR showed an increase in CHK2 phosphorylation levels suggesting that its function was actively modulated during DDR (Matsuoka *et al.*, 1998). At that time, the phosphatase CDC25 was already known for its role in regulating cell cycle progression (Walworth *et al.*, 1993; Furnari *et al.*, 1999). Considering the role of Rad53 in cell cycle checkpoints regulation, the authors demonstrated that CHK2 phosphorylated CDC25A, CDC25B and CDC25C. Using a kinase-dead allele from *CHK2*, the authors mapped a phosphorylation site at serine 216 of CDC25C, a site known to be involved in its negative regulation (Ogg *et al.*, 1994). Moreover, the kinase activity of CHK2 was shown to be dependent on treatment with UV, IR or HU suggesting that CHK2-dependent phosphorylation of CDC25C is modulated in response to DNA damage (Matsuoka *et al.*, 1998).

To test whether CHK2 function depended on signals elicited by upstream kinases, the authors evaluated the phosphorylation status and activity of CHK2 on A-T irradiated cells. The experiment indicated a reduction in CHK2 phosphorylation associated with a decrease in its kinase activity. Finally, the ectopic expression of *ATM* rescued both phosphorylation status and activity of CHK2, suggesting that ATM is located upstream to CHK2 in a signaling pathway responsible for regulating the cell cycle checkpoints (Matsuoka *et al.*, 1998) (Figure 2).

A few months before the identification of *CHK2*, Sanchez *et al.* (1997) used a degenerate PCR strategy and identified *CHK1*, a human gene very similar to *S. pombe chk1* (Table 1 and Figure 1). In parallel, *CHK1* was also isolated in Tony Carr’s laboratory (Flaggs *et al.*, 1997). Human CHK1 showed an increase in DNA damage-dependent phosphorylation, suggesting that like its yeast counterpart (Walworth and Bernards, 1996), its function was modulated during DDR. In addition, as observed for CHK2, CHK1 phosphorylated CDC25C at serine 216 (Sanchez *et al.*, 1997; Matsuoka *et al.*, 1998). This result demonstrated the existence of two DNA damage responsive kinases capable of promoting the same inhibitory signal in CDC25C. Despite the functional redundancy, it was suggested that CHK1 and CHK2 could play different roles depending on the type of damage elicited and/or the stage of the cell cycle in which they were active (Figure 2). It was also possible that they were specific for other unknown substrates. Interestingly, unlike CHK2 whose regulation was attributed to ATM-mediated signaling, which PIKK was responsible for regulating CHK1 in mammalian cells remained unknown. Eventually, through the use of conditional CHK1*-*deficient cell lines and a dominant negative mutant of ATR, it was demonstrated that the regulation of CHK1 was indeed dependent on ATR (Liu *et al.*, 2000) (Figure 2).

## *S. cerevisiae* Chk1, a DDR kinase involved in mitotic arrest

Based on the *S. pombe* and human sequences of *CHK1*, the *S. cerevisiae* ortholog was identified by similarity with the unknown ORF YBR274w (Figure 1). Unlike its human counterpart, *S. cerevisiae chk1* mutants were not essential to cell viability. Despite no evident alteration in the S phase checkpoint, *chk1* mutants synchronized with nocodazole, an inhibitor of microtubule polymerization, showed moderate sensitivity and defects in cell cycle arrest in response to IR treatment, suggesting a potential involvement of *Chk1* in the regulation of mitosis progression (Sanchez *et al.*, 1999). Chk1 was phosphorylated in response to DNA damage. Moreover, phosphorylation of both Chk1 and Rad53 was shown to be dependent on Mec1 but independent from each other (Sanchez *et al.*, 1999). These results indicated that both Rad53 and Chk1 were independently regulated by Mec1 (Figure 2). Further experiments showed that different from its *S. pombe* and human ortholog, the *S. cerevisiae* Chk1 promoted cell cycle arrest through a different mechanism involving the regulation of Pds1. Pds1, known as a Securin, prevents the segregation of sister chromatids thus inhibiting anaphase entry. By combining yeast genetics and biochemical approaches, Sanchez *et al* demonstrated that during DDR, the *CHK1*-mediated response promotes the stability of Pds1, thus contributing to cell cycle arrest prior to anaphase entry (Sanchez *et al.*, 1999).

The identification and functional characterization of *S. cerevisiae* Chk1 showed that, unlike Rad53, which promotes cell cycle arrest during G1/S, S phase and G2/M transitions, Chk1 promotes cell cycle arrest during mitosis. These observations suggested that Rad53 and Chk1 could be acting in parallel to reinforce DDR through a fail-safe mechanism that guarantees cell cycle arrest at different stages (Figure 2).

## A brief consideration on DNA-PK, a DDR kinase with no homologues in yeast

In 1986, the group of Carl W. Anderson accidentally discovered that linear fragments of dsDNA induced the phosphorylation of several proteins in extracts of widely divergent metazoan species (Walker *et al.*, 1985). In the following years, several laboratories identified the protein responsible for this kinase activity as DNA-PKcs (DNA-dependent protein kinase catalytic subunit), another member of the PIKK family. It was latter established that DNA-PK is recruited to DSBs by the heterodimeric Ku complex to promote DSB repair by non-homologous end joining (NHEJ). Therefore, in addition to ATM and ATR, mammalian DDR is also coordinated by DNA-PKcs (Blackford and Jackson, 2017). It is important to note that although yeast presents all core NHEJ factors, it lacks the catalytic DNA-PKcs. In this case, other factors such as Mre11, Rad50 and Xrs2 (MRX complex), may compensate for the lack of DNA-PK.

## DDR kinases in the 21^st^ century: advances and perspectives

Since the discovery of the DDR kinases, the identification of their substrates progressed slowly with only a few targets being identified during the late 1990s and early 2000s. However, over the last decade, technical advances in phosphoproteomics had a profound impact in the DDR field, expanding the identification of proteins phosphorylated by the DDR kinases in both yeast and human cells (Beausoleil *et al.*, 2004; Olsen *et al.*, 2006; Albuquerque *et al.*, 2008). Furthermore, the advent of quantitative mass spectrometry analysis allowed researchers to monitor the dynamics of DNA damage signaling by looking simultaneously at multiple DDR kinase substrates in a systematic and unbiased manner (Bastos de Oliveira *et al.*, 2015; Willis *et al.*, 2016; Zhou *et al.*, 2016; Lanz *et al.*, 2018; Bass and Cortez, 2019). For instance, a recent study based on quantitative phosphoproteomics showed that the human DDR activators ETAA1 and TopBP1 regulate distinct aspects of ATR signaling. By monitoring ATR-dependent phosphorylation events in ETAA1 and/or TopBP1 deficient cells, the authors revealed that while TopBP1 is the primary ATR activator of replication stress, ETAA1 coordinates ATR signaling during mitosis (Bass and Cortez, 2019).

Recently, the advent of genome-editing tools in human cell lines, combined with new DDR kinase inhibitors, have been successfully applied for the screen of synthetic lethal interactions, offering new insights for cancer treatment (Ruiz *et al.*, 2016; Gerhards and Rottenberg, 2018; Wang *et al.*, 2019). The kinase ATR, for example, have come under the spotlight in recent years as prominent therapeutic target in cancer (Foote *et al.*, 2015). In order to cope with high levels of replication stress, cancer cells depend on ATR for survival and proliferation (Choi *et al.*, 2011; Toledo *et al.*, 2011a). Therefore, pharmacological inhibition of ATR selectively sensitizes different types of tumor cells, especially in tumors with defects in the ATM-p53 pathway (Charrier *et al.*, 2011; Peasland *et al.*, 2011; Reaper *et al.*, 2011; Toledo *et al.*, 2011b; Fokas *et al.*, 2012; Foote *et al.*, 2013, 2015, 2018; Kim *et al.*, 2018).

The knowledge accumulated over the last three decades, since the discovery of the first DDR kinase in yeast, was fundamental to our understanding of how cells coordinate the multiple responses that confer protection against genomic instability (Ciccia and Elledge, 2010). Moreover, besides their importance for genome integrity, it has been suggested that DDR kinases regulate other biological processes such as protein homeostasis, carbon and phosphatidylinositol metabolism, vesicle trafficking, and autophagy (Simpson-Lavy *et al.*, 2015; Zhou *et al.*, 2016; Dahl and Aird, 2017; Yi *et al.*, 2017; Cheng *et al.*, 2018; Corcoles-Saez *et al.*, 2018, 2019). However, it remains to be established the relevance of these processes during DDR. Finally, the recent discovery that DDR kinases are activated by oncogenic stress (Halazonetis *et al.*, 2008) put them as promising targets for clinical applications. Thus, whether for the basic or translational research aspect, there is plenty of space to continue exploring the biology of DDR kinases in both yeast and humans.
